# The evolution of a coordinator from a vocative source: the case of the disjunctive *ja:* in Jordanian Arabic

**DOI:** 10.1016/j.heliyon.2021.e08505

**Published:** 2021-12-01

**Authors:** Abdulazeez Ahmad Jaradat, Mohammad Anwar Al-Taher

**Affiliations:** Applied Science Private University, Jordan

**Keywords:** Grammaticalization, Bisyndetic disjunction, Coordinator, Vocative, Proximity, Syntactic distribution

## Abstract

This research paper proposes that a vocative can be a potential source of coordination, thus it adds to the literature on the grammaticalization of coordinators. Evidence to this proposal is taken from Jordanian Arabic (JA) wherein the vocative particle *ja*: developed a disjunctive (coordination) function. The synchronic evidence to the evolution of the disjunctive *ja:* is that this function is conveyed by this particle in some but not all Arabic varieties, whereas the vocative function is the common cross-dialectal function of *ja:*. Further, this study suggests that the factors that licensed the development of this function (i.e., the disjunctive *ja:*) in JA are (i) the common semantic feature between the vocative and disjunctive *ja:*, namely proximity, (ii) the shared function of warning and (iii) the syntactic distribution of *ja:* in initial position of the two conjuncts of the bisyndetic disjunctive construction. With regard to the properties of this evolution, it is demonstrated in this paper that the development of the coordinating *ja:* is a case of secondary grammaticalization featured by expansion in functionality and increase in syntactic contingence of the hosting structure.

## 1 Introduction

Coordinating constructions contain at least two conjuncts.^1^ They typically branch into syndetic and asyndetic coordinating constructions (Haspelmath 2004, 2007). The former has at least one coordinator between conjuncts, such as the English *and*, *but* and *or*, whereas the latter exhibits no coordinators.^2^ Syndetic coordination branches further into monosyndetic and bisyndetic. A mono-syndetic construction involves only one coordinator, whilst a bisyndetic construction has two coordinators or a replicated coordinator. The main concern of the current study is the bisyndetic disjunctive coordinating construction *ja: X ja: Y* ‘either X or Y’ in Jordanian Arabic (henceforth JA), an Arabic vernacular variety. It is to demonstrate that *ja:*, which is commonly a vocative particle in Arabic varieties, performs another function in JA, namely disjunctive function.^3^ This implies that the vocative *ja:* was grammaticalized into a disjunctive coordinator from a vocative source in JA.

Bisyndetic coordinating constructions, such as the positive *either X or Y* and the negative *neither X nor Y* in English, are cross-linguistically common and have various manifestations (Haspelmath 2004, 2007). In Standard Arabic (SA), positive bisyndetic coordination is expressed by *ʔimma X wa ʔimma Y* (Adeemah 1984), which is somehow equivalent to the English *either X or Y*, as shown in (1).

**Figure:**
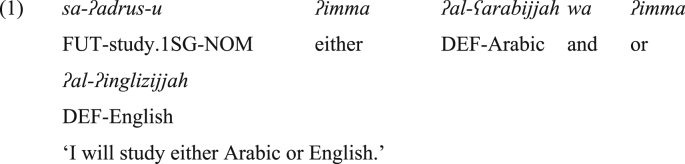

On the other hand, the typical vocative particle *ja:* in Arabic acquired a new grammatical function in JA. It serves as a disjunctive coordinator in the positive bisyndetic coordinating construction *ja: X ja: Y* ‘either X or Y’ in JA, as exemplified in (2), and thus it is somehow equivalent to the positive bisyndetic coordinating construction *ʔimma X wa ʔimma Y*, which is the hallmark of positive bisyndetic coordination in SA.^4^

**Figure:**
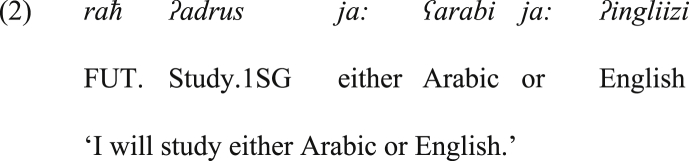

The main research question of the current study is: how did the vocative *ja:* develop a disjunctive function in JA? The significance of this study is in the observation that the development of coordinators into other functional categories, such as discourse markers and final particles, is one of the main concerns in language change and grammaticalization studies (e.g., Barth-Weingarten and Couper-Kuhlen 2002; Hengeveld and Wanders 2007; Mulder and Thompson 2008; Hancil 2014, among others); however, the evolution of coordinators themselves has less attention. The current study is an investigation to the grammaticalization of the vocative *ja:* into a coordinator in the bisyndetic disjunctive construction.

Evidence to this grammaticalization path in this study is synchronic. To illustrate, the vocative particle and its disjunctive counterpart share a common semantic ground, nearness or proximity, and they have a common function, namely warning. This implies that one of these two grammatical items can be the source of its counterpart. In this paper, we suggest that the vocative particle *ja:* is the source of the grammaticalized coordinating *ja:*, as the disjunctive function of *ja:* is found in some but not all Arabic varieties, unlike its vocative function. On this ground, this study adds to the relevant literature of grammaticalization by proposing that a vocative particle is another source of coordinators, next to the categories reported in other studies on various languages, such as adverbs and prepositions. Beside the shared semantic feature and function, another factor that paved the way to the coordinating function is a syntactic one; the syntactic distribution of *ja:* in the initial position of the two conjuncts of the disjunctive bisyndetic construction licensed its inheritance to the disjunctive function.

The grammaticalization path proposed in this study is vocative particle → disjunctive coordinator; however, the full grammaticalization path that encompasses the evolution of the vocative *ja:* is beyond the scope of the current study, as it requires diachronic evidence. To put it in other words, it cannot be determined in this study whether the source of the vocative particle is, for example, tune-driven or a conative interjection, as reported about the evolution of vocatives in other languages (Andersen 2012; Sóskuthy and Roettger 2020).

Additionally, it is argued in this paper that the grammaticalization of the coordinating *ja:* is a case of seconday grammaticalization. In the relevant literature of secondary grammaticalization, it is reported that there are two subtypes of secondary grammaticalization. The former results in morpho-phonological reduction and increase in morpho-syntactic bondedness (Traugott 2002; Norde 2019), while the latter is characterized by expansion in functionality (Detges and Waltereit 2002; Kranich 2010; Waltereit 2011; Breban 2014). This study demonstrates that the secondary grammaticalization of *ja:* is a case of expansion in functionality paired with increase in contingence on the surrounding syntactic structure (i.e., the syntactic distribution of the disjunctive coordinator is very restricted, unlike the vocative counterpart).

The current paper is outlined as follows: Section 2 proposes that *ʔimma* is a coordinator within the bisyndetic disjunctive coordinating construction in SA, whereas *ja:* serves this function in JA. Section 3 is to review the grammaticalization paths of conjunctions from a cross-linguistic point of view. It is also to show that there is a semantic and functional ground shared by the vocative and the disjunctive function of *ja:* and to argue that the syntactic distribution of *ja:* in the bisyndetic disjunctive construction paved the way for its evolution into a disjunctive coordinator. Section 4 hints on the grammaticalization of the vocative *ja:*. It speculates that this vocative particle is either from a tune-driven source or from a conative source. In Section 5, we demonstrate that the grammaticalization of the disjunctive *ja:* in JA is a case of secondary grammaticalization involving expansion in functionality with increase in contingence on the surrounding syntactic structure. Section 6 is to conclude.

## 2 Emphatic coordination in Arabic

### 2.1 Emphatic coordination in Standard Arabic

First, it should be highlighted that the current study relies on the following data sources: the intuition of the researchers as native speakers of JA and naturally occurring data elicited from Twitter and Facebook free speech and JA television series. With regard to SA, the SA data were collected from various Arabic grammar books.

As shown in (1) above, SA has a bisyndetic disjunctive coordinating construction, namely, *ʔimma X wa ʔimma Y*. This construction serves a positive function, and offers alternatives from which the hearer obligatorily will choose. It is also emphatic, as it emphasizes that each conjunct belongs to the coordination, ‘and each of them is considered separately’ (Haspelmath 2007: 15). Therefore, this construction is expected to occur in contexts wherein the hearer obligatorily chooses one of the alternatives. On the other hand, a non-emphatic construction that has the shape of *X or Y* is not necessarily exploited in such a context. The construction *ʔimma X wa ʔimma Y* comprises two instances of *ʔimma* at the left of each conjunct, and the second *ʔimma* is preceded by the conjunctive *wa* ‘and’.^5^ Most Arab grammarians propose that both *ʔimma*'s are adverbials. They are to introduce details (or alternatives), and *wa* to the left of the second *ʔimma* is the only coordinator in the construction. However, this proposal seems inadequate. This inadequacy is a consequence of the interpretation of the construction. The whole construction yields disjunctive coordination, but not conjunctive coordination. In other words, it offers alternatives, and the addressee must choose one. On this ground, it can be assumed that *ʔimma* in this construction should be treated as a disjunctive coordinator, yet it cannot stand alone without the conjunctive coordinator *wa* to the left of the second instance of *ʔimma*. On the other hand, if *ʔimma*'s are deleted in the construction, this renders the target sentence lacking the disjunctive (and the emphatic) meaning. On this basis, it is suggested in this research paper that *ʔimma* is a disjunctive coordinator, and this is why the interpretation that surfaces is the disjunctive one.

An important point that should be raised here is that the coordinator *wa* ‘and’ is mandatory with *ʔimma* at the left of the second conjunct, as in (1) above. This may imply that *ʔimma* is not related to coordination, as excluding the conjunctive *wa* leads to ungrammatical structure. However, the presence of *wa* and the absence of *ʔimma* changes the type of coordination from disjunctive to conjunctive, and in the presence of both (*wa* and the two instances of *ʔimma*), only the disjunctive reading can surface (never the conjunctive one). Hence, it seems that the conjunctive *wa* is grammatically necessary and for a large extent has nothing to do with the semantics of coordination. From a semantic perspective, *wa* only brings the two alternatives together. Whereas the scope indicator *ʔimma* (the first one) and the disjunctive coordinator *ʔimma* (the second one) are the particles that give rise to the disjunctive and emphatic meaning. On this basis, we suggest that *wa* and the second instance of *ʔimma* together form the disjunctive coordinator, which is equivalent to the English *or*, whereas the first instance of *ʔimma* alone is the scope indicator. Note that we will show that the disjunctive *ja:* in JA is more independent than *ʔimma* in SA, as it blocks the occurrence of *wa*.

Now, what supports the proposal that the construction in (1&3) belongs to the domain of emphatic bisyndetic coordination (neither monosyndetic nor polysyndetic) is its interpretation and its incompatibility with multiple coordination (i.e., more than two coordinated phrases/sentences). To illustrate, the speaker in (3b) restricts his/her options to two languages. This strongly indicates that the speaker is not open to study any other language. This restriction is the cause of ill-formedness in (3c) where the emphatic coordinating construction *ʔimma X aw ʔimma Y* is followed by *wa ʔimma Z*, or even *ʔaw Z* ‘or Z’, as marked by an asterisk. This is at odds with the example in (3d) wherein the absence of both *ʔimma*'s implies that the speaker would like to study one of these languages, but this does not rule out the possibility that he/she may consider studying another language. Therefore, the insertion of *ʔaw Z* after *X ʔaw Y* does not lead to ill-formedness. This implies that the sentence is not emphatic and not necessarily bisyndetic in the absence of the two *ʔimma*'s.

**Figure:**
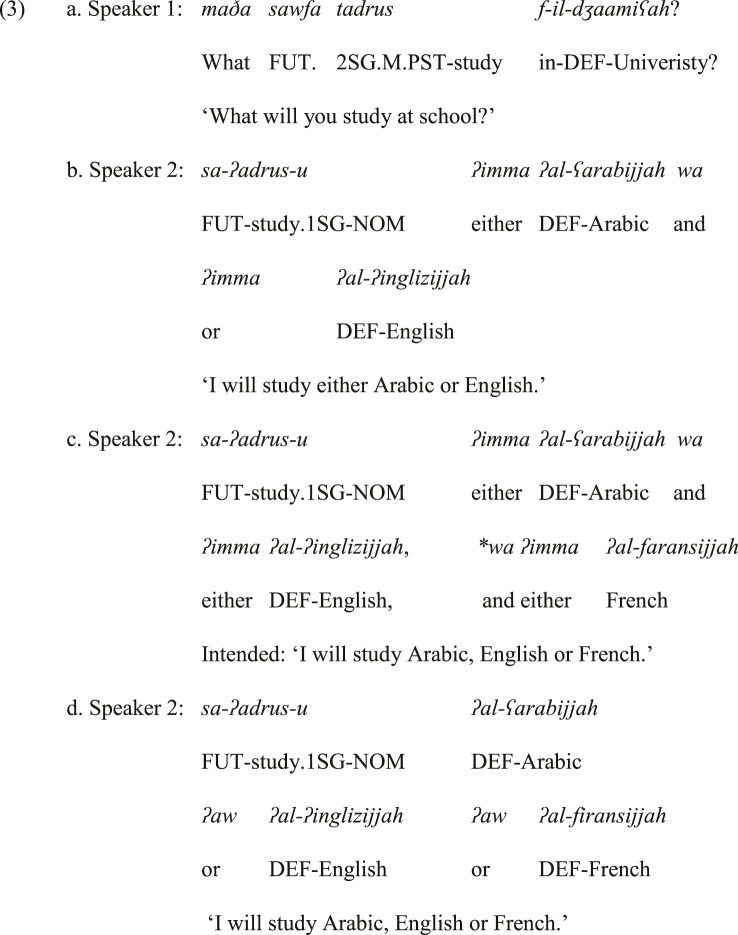

Hence, the constructions *ʔimma X wa ʔimma Y* and *X ʔaw Y* are to coordinate options; however, the former is always emphatic and bisyndetic, whereas the latter is not or less emphatic and can be bisyndetic.

Here, it is worth discussing the status of the conjuncts within the construction *ʔimma X wa ʔimma Y*. This construction is not category-sensitive. To exemplify, the two conjuncts of the construction *ʔimma X wa ʔimma Y* are typically phrases, such as the nominals (arguments) in (4a), the adjectival phrases (adjectival predicates) in (4b) or the prepositional phrases in (4c). It is also common that each *ʔimma* is followed by a conjunct that constitutes a full sentence preceded by the infinitive *ʔan* ‘that’, as in (4d).

**Figure:**
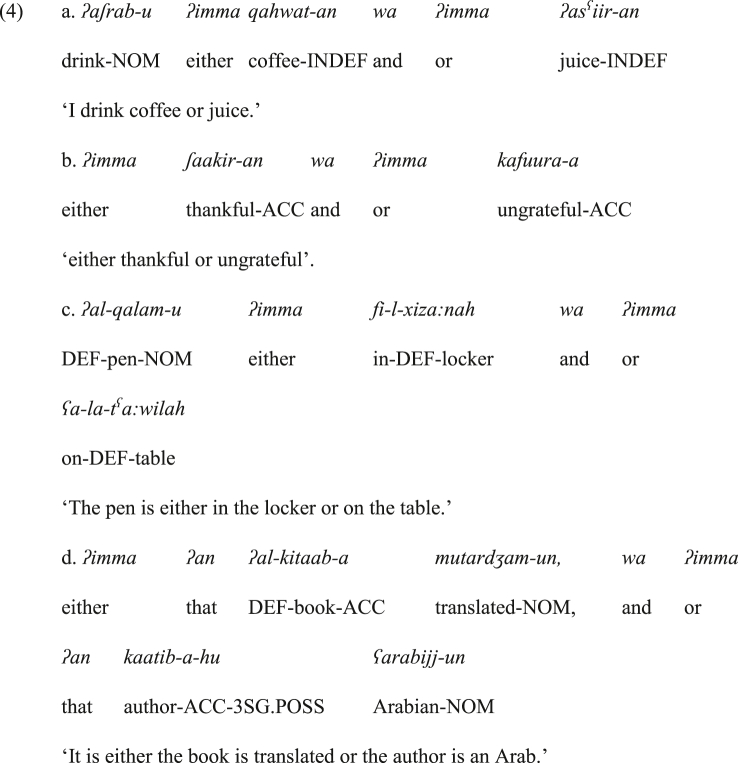

In this part, it has been argued that the coordinating construction *ʔimma X wa ʔimma Y* in SA has the following characteristics: positive, emphatic, disjunctive and bisyndetic. In addition, it has been proposed that *ʔimma* is a true disjunctive coordinator in SA, contrary to the view of some Arab grammarians. Below, it will be shown that this coordinating construction is not fully preserved in JA, as *ʔimma* is replaced with the grammaticalized disjunctive coordinator *ja:*.

### 2.2 Emphatic coordination in Jordanian Arabic

In JA, the emphatic bisyndetic coordinating construction *ʔimma X wa ʔimma Y* is neither the common positive emphatic construction, nor an obsolete construction. In this variety, this form underwent some modifications.^6^ Let us say that it has more than one variant. The possible variants in the variety are: (a) *ja: ʔimma X ja: ʔimma Y*, (b) *ja: ʔimma X ja: Y* and (c) *ja: X ja: Y*, as shown respectively in (5). Hence, both varieties, SA and JA, make use of *ʔimma*, yet its presence is optional in JA and obligatory in SA in the target coordinating construction. Further, the conjunction *wa* ‘and’, which is obligatory in this construction in SA, is not attested in JA. Alternatively, *ja:* is the typical particle used to the left of *ʔimma*.

**Figure:**
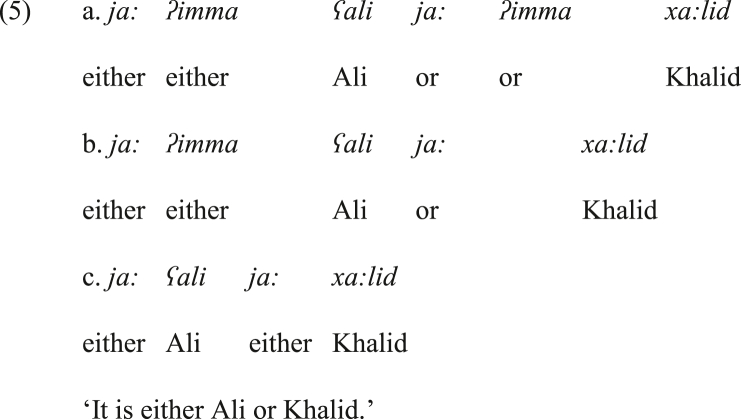

The variants of the disjunctive coordinating construction in (5a&b) are attested in JA; however, they are far less frequent than the construction in (5c) in the current form of JA. In other words, the presence of *ʔimma* to the left of the conjuncts is not common. On the contrary, the occurrence of *ja:* at the left of each conjunct is obligatory.

Here, we should report that the typical function of *ja:* is not coordinating in Arabic. It is rather the most common vocative particle in this language and its varieties. On this ground, the current paper argues that *ja:* has been developed into a disjunctive coordinator used only in the positive emphatic bisyndetic coordination in JA, unlike *ja:* in SA where it can only be used as a vocative particle. For exemplification, the only possible interpretation of the structure from SA in (6) is the vocative sense (i.e., *ja:* cannot be considered a coordinator). This implies that the functional item *ja:* in JA has gained a new grammatical function that is not attested in SA, which is coordination.

**Figure:**
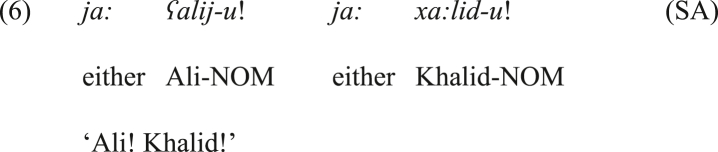

On the other hand, *ja:* can be either treated as a vocative particle or a coordinator. The variants in (5) indicate that the disjunctive coordinator in JA was compatible with *ʔimma* in the same construction, but in the current form of JA the co-occurrence of *ʔimma* and the disjunctive *ja:* is the marked form. In turn, this implies that the disjunctive *ja:* is a grammaticalized marker that replaced *ʔimma* in JA. Further, the data in (5) suggest that the bisyndetic disjunctive construction *ja: X, ja: Y* replaced the older variant *ʔimma X, ʔu ʔimma Y*, and in between creating a stage of real superfluousness in this context, until *ja: X, ja: Y* became more dominant, as it currently is. Then, the superfluous structure *ja: ʔimma X, ja: ʔimma Y* is predicted to be dropped. What supports this prediction is that the structure in (5b), where the second instance of *ʔimma* is dropped, is more frequent and less marked than the full structure in (5a) in JA.^7^ To put it differently, dropping the second *ʔimma* is an indicator to the potential dropping of the first *ʔimma* by time.

Here, it is of great importance to determine whether the first or the second interpretation is intended in a particular sentence/utterance *hosting ja:* in JA. The intended reading can be idenitifed by referring to the intonational structure of the utterance and some temporal cues (e.g., final lengthening). Further, prosodic/metrical (non-)reduction is also a crucial diagnostic to the nature of *ja:*, whether it is a vocative particle or a coordinator. Regarding vocatives, it is somehow frequent cross-linguistically to have an easily perceived final lengthening at the end of each vocative phrase, the main pitch accent on the addressee, and a sustained (plateau) tone to the end of the vocative phrase (Gussenhoven 1993; Ladd 2008; Borràs-Comes et al., 2015). On the other hand, the entire utterance is a positive bisyndetic emphatic disjunctive coordinating construction, and *ja:* is a disjunctive coordinator in JA, if the following conditions (especially the first two conditions) are met. The first part of the sentence ends with a rising tone, which is a common non-final tone to indicate continuity (see Truckenbrodt 2004; Kawahara and Shinya 2008 for more details of continuation tune). This continuation tone implies that the second alternative of the coordinating construction (i.e., the second conjunct) has not been uttered yet. *ja:* is produced in its full form (unlike the vocative one). Sentence-final position is characterized with a considerably less prominent lengthening (i.e., it can be barely perceived). On this basis, the vocative *ja:* is subject to phonological reduction, whereas it resists such reduction as a disjunctive coordinator.

Another piece of evidence supporting the disjunctive coordinating function of *ja:* in JA is that it does not co-exist with the conjunctive coordinator *ʔu* ‘and’ (a vernacular variant of the standard *wa*). In (7a), the cause of ungrammaticality is the co-existence of *ʔu* and *ja:* to the left of the second conjunct. The former gives rise to conjunctive interpretation, whereas the latter denotes disjunction. Therefore, the semantics of the structure crashes. To render this structure grammatical, the conjunctive *ʔu* must be deleted. The deletion of the second *ja:* will not resolve the problem as the first disjunctive *ja:* is also semantically at odds with the conjunctive *ʔu*. Moreover, the common disjunctive monosyndetic coordinator *ʔaw* ‘or’ cannot be inserted to the left of the second *ja:*, as shown in the ungrammatical structure in (7b). The source of ungrammaticality is having two different disjunctive coordinators (*ʔaw* and *ja:*) that serve the same function (i.e., disjunction) to the left of the second conjunct. The inability of the monosyndetic *ʔaw* to participate in the nature of this structure implies that *ja:* is a true bisyndetic disjunct in JA.

**Figure:**
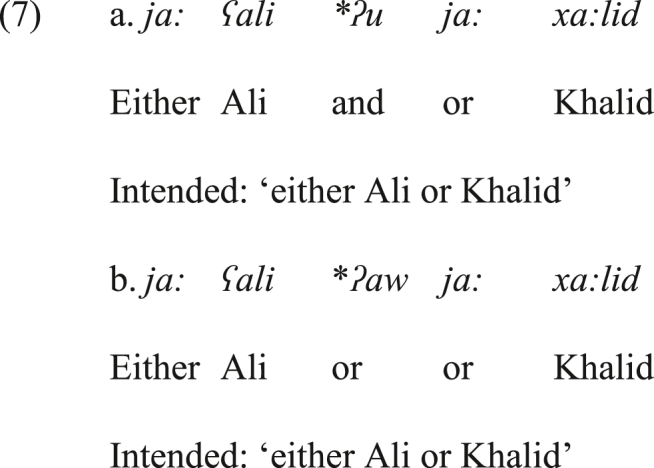

It should be admitted here that *ja:* in JA is a more obvious case of disjunctive coordination than *ʔimma* in SA, as the second *ʔimma* at the left of the second conjunct in a bisyndetic construction obligatorily, unlike *ja:*, requires the presence of the conjunctive coordinator *ʔu*, which should be treated as an obligatory part of the disjunctive *ʔu ʔimma* to the left of the second conjunct.

To conclude, the functional item *ja:* in this subsection has been introduced as a disjunctive coordinator that occurs in the coordinating construction which has the following characteristics in JA: positive emphatic bisyndetic and disjunctive. The remaining question is how it acquired this grammatical function. In the following section, a scenario to the grammaticalization of the disjunctive coordinator *ja:* in JA is suggested. It will be proposed that *ja:* inherited the emphatic disjunctive function from *ʔimma* in JA, and it no longer denotes the vocative reading in the context of disjunctive coordination in the current form of JA.

## 3 The source of the disjunctive *ja:*

This section is divided into three subsections. In 3.1, we review the grammaticalization paths of conjunctions from a cross-linguistic viewpoint. In 3.2, the main semantic feature and function shared by the vocative and the disjunctive function of *ja:* are discussed. In 3.3, it is explained how the syntactic distribution of *ja:* in the bisyndetic disjunctive construction paved the way for its evolution into a disjunctive coordinator.

### 3.1 Grammaticalization of coordination: A cross-linguistic view

Relying on data from a number of languages from different language families, Mithun (1988) claims that the grammaticalization of conjunctions is recent in the languages of the world, and their emergence is parallel with literacy. In the relevant literature, some attention is paid to explore the source of coordinating conjunctions (syndetic); however, more focus is given to the late acquisition of conjunctions to more functions (e.g., the grammaticalization processes from coordination to transition and from coordination to succession (Qinghua 2007)) and to the development of subordinating conjunctions. To exemplify some, it is well documented that a complementizer can evolve from a demonstrative source, such as the English *that* and the German *das*, or from a case marker, such as the accusative case morpheme *wəə* in the Tungusic language Evenki. The source can also be prepositional (e.g., *for* in English) (by Hopper and Traugott 2003) or nominal (the development of the concessive *while* from the Old English lexical source that mean *at the time that* (Lee 2006)).

Despite the scarcity of studies on the origin of coordinating conjunctions, a common source of conjunctions reported in the literature is adverbs, such as the development of the conjunctive *paː* ‘and’ from the adverb *pɐːnə* ‘otherwise’ in the Uralic language Khanty (Borise and Kiss 2021). Another example is the Mandarin Chinese *buguo* ‘only’ in clause-non-initial position that is used as an adversative conjunction in clause-initial position in (8b). Noteworthy is that the cause of its acquisition of conjunctive status is its ability to move to clause-initial position, unlike some other adverbs that cannot move to this position.

**Figure:**
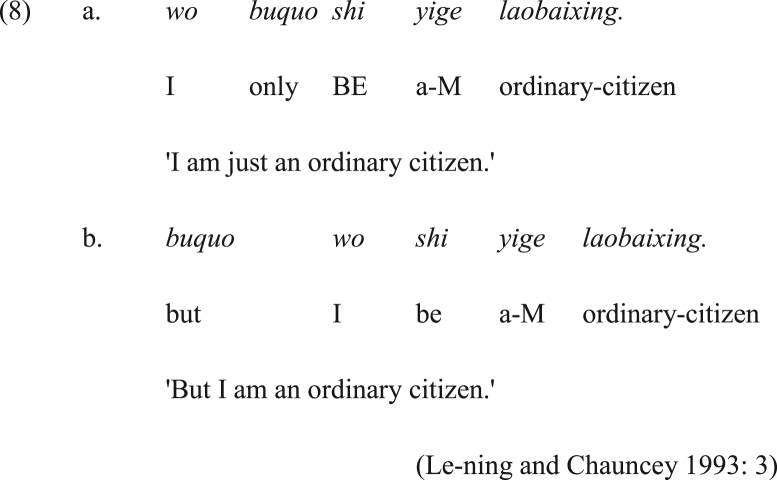

Further, coordinating conjunctions, especially adversatives, may have nominal sources, such as the evolution of the noun *álà* ‘God’ (borrowed from Arabic Allah) into an adversative conjunction similar in meaning to *but* (Frajzyngier 1996). Frajzyngier (1996) suggests that the grammaticalization path of this conjunction is as follows: the noun *álà* → exclamation marker → adversative conjunction. Additionally, it is reported that conjunctions can be evolved from a prepositional source and (auxiliary) verbal source, such as the Chinese conjunction *gong* whose grammaticalization path started with the evolution of a verbal source into a preposition, and then the proposition was turned into a conjunction.

Qinghua (2007) suggests that there are a number of common sources to conjunctions in the languages of the world. The most common source of conjunctions is words that mean togetherness, such as the togetherness preposition *mai* in Wa language. Less commonly, words that mean sameness might develop into conjunctions in some languages. For example, *loŋ*, a copula that denotes sameness, is evolved into a conjunction in Lhoba, a Tibetan-Myanmese language family. Besides, words that denote nearness develop into conjunctions in some languages, such as Chinese *jí*, which means *to reach* and the Near (proximal) deictic *zhè* are used as coordinating conjunctions.

### 3.2 The semantic resemblances between the vocative and the disjunctive *ja:*

In Section 2, it has been introduced that the disjunctive coordinator *ja:*, which is used in the positive emphatic bisyndetic construction in JA, is originally a vocative particle. What emphasizes the proposal that the source of the coordinating *ja:* is the vocative counterpart is that the predominant and prominent function of the functional item *ja:* in all Arabic varieties is the vocative one, as shown in the SA and JA examples in (9a&b), whilst its disjunctive function at best is less common. Its disjunctive function is not available in SA.^8^ *ja:* in SA is always interpreted as a vocative particle and cannot be treated as a disjunctive coordinator, as noted in the previous section, whereas it can be a disjunctive coordinator in JA.

**Figure:**
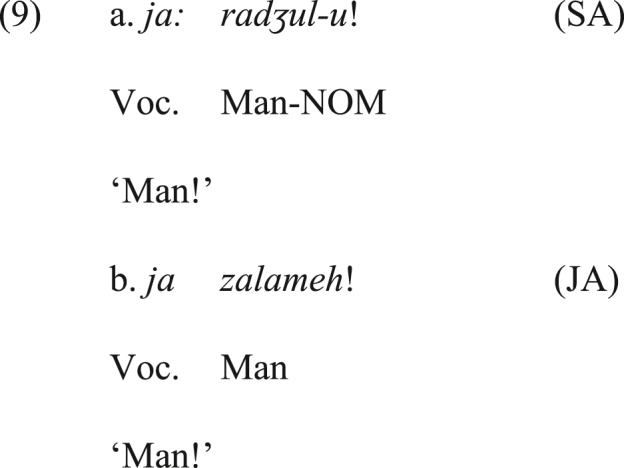

The main proposal in the current study is that the vocative *ja:* was developed into a disjunctive coordinator in JA. The grammaticalization path of the disjunctive *ja:* is as follows: vocative particle → a disjunctive coordinator (in bisyndetic constructions), but why did it acquire this grammatical function in JA? More precisely, what is the meaningful relation between the vocative and the coordinating function?

Let us first give some examples on the naturalness of grammaticalization. Grammaticalization, which forms grammar by the gradual shift from the lexical domain to the functional domain, or the shift within the grammatical domain, occurs in paths (Lehmann 1995 [1982]) that may recur in many languages (or even several language families). To exemplify some, it is cross-linguistically common to develop futurity-denoting modal verbs from motion verbs (Bybee et al., 1991; Maisak 2002; Jarad 2014), to develop discourse markers from conjunctions and to assign the progressive aspect to a posture verb, such as the Arabic posture verb *ga:ʕid* ‘sitting’ (Maisak 2002; Camilleri and Sadler 2017). Further, pronouns, beside deictic particles and existential verbs, are a common source to copulas in natural languages (Pustet 2003; Camilleri and Sadler 2019). In all these examples, the meaningful (metaphorical) link between the source of grammaticalization and the newly assigned function to the source word seems to be clear. To illustrate some, it is natural to assign the meaning of futurity to a motion verb, as the motion verb involves the movement spatially from one a starting point to a certain destination. Likewise, talking about futurity refers to two different points on a temporal scale. Thus, the grammaticalization futurity marker from a motion verb denotes the transition from the moment of talking to a certain point of time in the future. It is also natural to develop an auxiliary verb denoting progression from a posture verb that means *sitting*, as the meaning of setting encompasses progression and continuity (i.e., sitting for some time). Another clear connection is that between a pronoun and a copula. A pronoun may develop into a copula, as they have a common function, namely linking between two things. A pronoun links a person, object or abstract concept in the real world to the world of the host utterance. Likewise, a copula connects between a nominal and an adjective in a predicational sentence, or between two nominals in an equational sentence.

The previous discussion implies that grammaticalization paths are commonly viewed as natural processes that occur regularly in many natural languages, as their triggers are natural and explainable. Now, the question that is crucial to the current study is: what is the logical or meaningful connection between the vocative *ja:* and its disjunctive coordinating counterpart? In other words, how did it acquire this disjunctive function? Based on Qinghua (2007) descriptive analysis to the potential grammaticalization paths of conjunctions, it seems obvious that the vocative source *ja:* expresses nearness. More specifically, a person who uses the vocative *ja:*, intends to ask the addressee to be closer spatially or perceptually by attracting his/her attention. In other words, such markers (either segmental or non-segmental/tunes) ‘are often used to bridge physical distances between interlocutors’ (Sóskuthy and Roettger 2020:3). Thus, we suggest that this is the motivation of the evolution of the vocative particle *ja:* into a disjunctive coordinator. The proximity, which is a demand once the vocative particle is uttered, made the vocative particle *ja:* an optimal candidate to acquire the coordinating function later. The logic behind the acquisition of words that denote or require proximity into coordinators is that coordinators also express proximity. More specifically, they create a link between specific conjuncts (e.g., phrases or sentences).

To elaborate more on the semantic connection between the vocative and coordinating function, vocatives should be explained first. Vocatives can be described as a means for calling ‘the attention of an addressee, in order to establish or maintain a relationship between this addressee and some proposition’ (Lambrecht 1996: 267). Hence, the vocative *ja:* and its disjunctive counterpart in JA have a common denominator from a semantic perspective. To clarify, one function of the vocative *ja:*, just like vocative particles in natural languages, is to attract the attention of the addressee. Additionally, it can be used as a sign of warning to alert hearers especially when the vocative phrase is uttered with a high rising tone, which is a common intonational pattern of imperatives (Truckenbrodt 2006). For exemplification, in (10a) Speaker 1 decides that he will never get vaccinated, and Speaker 2 in (10b) is warning him how dangerous his decision is. Speaker 2 starts his turn with the vocative phrase *ja: zalameh* that should be uttered with a high rising intonation to denote warning.

**Figure:**
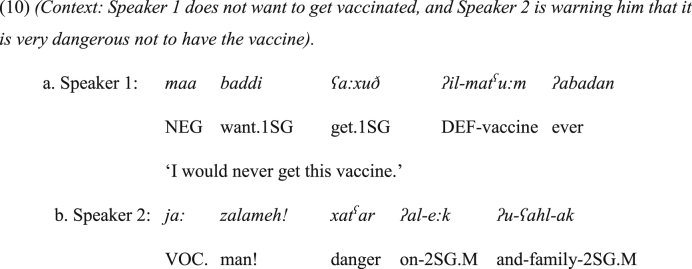

Additionally, some idiomatic vocatives in JA are normally used to warn the addressee. Consider the idiomatic vocative phrase in (11), which is to warn the hearers.

**Figure:**
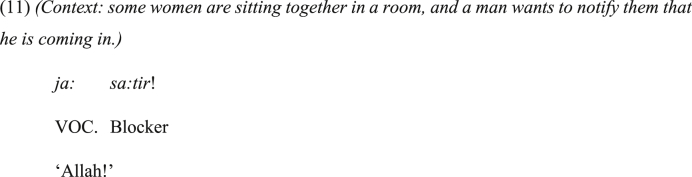

With respect to *ja:* as a disjunctive coordinator, as shown in (12), it has some meaning that is found in the vocative *ja:*. To illustrate, *ja:* in (12) is initially located to the left of each instance of *ʔimma* to attract the attention of the listener and add more emphasis to the disjunctive function of *ʔimma*. Note that at this stage of argumentation, *ja:* is left with no gloss to indicate that this functional item was not a proper disjunctive coordinator when it was initially introduced in the bisyndetic disjunctive coordinating structure.

**Figure:**
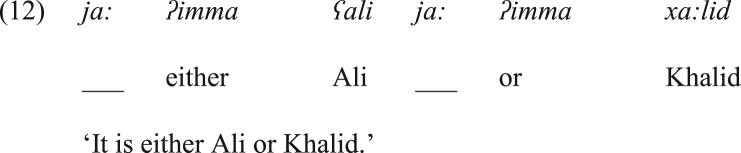

Further, it is common in JA to use the disjunctive *ja:* to warn the addressee. This should be a common and natural tendency, as offering alternatives for the hearer gives the impression that the speaker is warning him/her. In (13), for example, the father is warning his son by using the positive emphatic disjunctive bisyndetic construction containing *ja:*.

**Figure:**
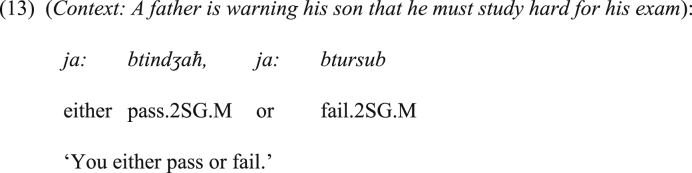

Based on the previous discussion, it seems plausible to propose that the source of the disjunctive coordinating *ja:* is the vocative one in JA, as they both have some resemblances at the semantic and functional levels, namely the meaning of proximity and the function of warning.

### 3.3 The syntactic distribution as a trigger to the disjunctive function

In this part, the addressed issue is why JA developed the bisyndetic disjunctive *ja:*, given that *ʔimma*, which has been introduced in Section 2 as bisyndetic disjunctive coordinator in SA, does exist in this dialect. Moreover, why can they co-occur in the same construction? This is ostensibly against the principle of language economy. Here, we suggest that the existence of *ja:* and *ʔimma* in the same construction at the first place in JA was purposeful. More precisely, *ja:* precedes each instance of *ʔimma* in order to attract the attention of the listener, as assumed above. The structure in (13), which is permitted in JA, can be taken as evidence that the introduction of *ja:* to the left of *ʔimma* was formerly to attract the attention, not to perform a disjunctive coordinating function. This function was rather performed by *ʔimma*.

**Figure:**
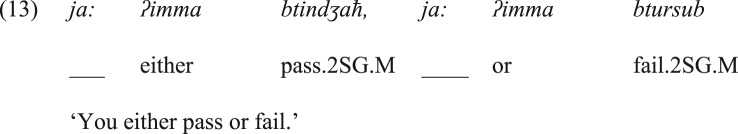

We further propose that by time, it was possible to drop *ʔimma* from the second conjunct, and then to drop it optionally from both conjuncts, as shown in (14a & b), respectively. Note that in the following examples, *ja:* is glossed as ‘either’.

**Figure:**
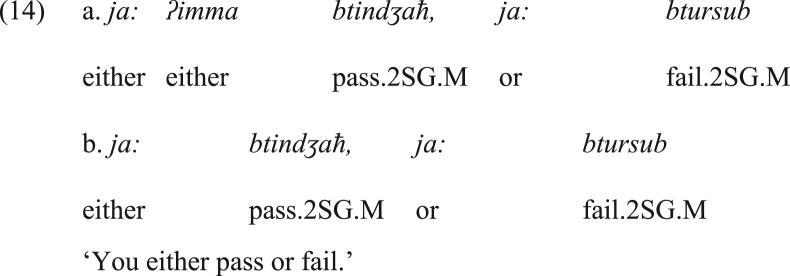

If the proposed scenario is borne out, the introduction of *ja:* to attract hearer's attention at the first place does not violate the principle of language economy. However, what is the trigger of dropping the disjunctive coordinating *ʔimma* from both conjuncts in JA? This optional dropping indicates that the disjunctive coordinating function is no longer a property of *ʔimma*. It is rather a feature of *ja:* by inheritance (i.e., it inherited this function from *ʔimma*), as they are adjacent elements. What supports this proposal is that in the current form of JA, *ʔimma* (or even its two instances) can be dropped form the disjunction construction, as in (14), whereas *ja:* cannot be deleted, as shown in the ungrammatical structures in (15). It is worth repeating here that the structure in (14b) (the structure lacking *ʔimma*) is the most frequent in JA. This implies that JA is on the track of obligatorily dropping *ʔimma* from its bisyndetic disjunctive construction, and this is compatible with the principle of language economy where one of the two grammatical items that perform the same function should be dropped.

**Figure:**
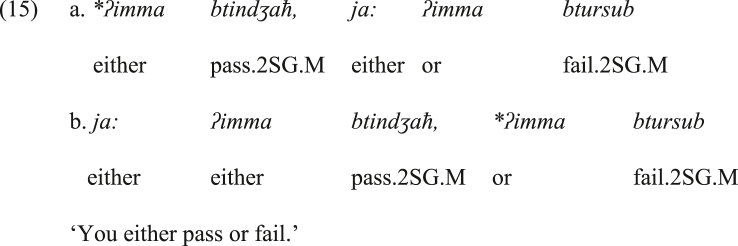

The previous discussion implies that *ja:* has been grammaticalized into a disjunctive coordinator, and the suggested path of its grammaticalization is as follows:

**Figure:**
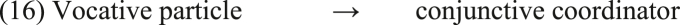

In this section, it has been shown that the triggers of this grammaticalization of the disjunctive *ja:* in JA are the shared meaning of proximity and the function of warning with the vocative counterpart. Further, its distribution in a disjunctive construction-initial position paved the way for the acquisition of this function. It initially entered a disjunctive construction to attract the attention and warn the hearer. Then, it inherited the disjunctive function from *ʔimma*. At the present stage, *ja:* as a replacement of *ʔimma* is very frequent in JA; however, *ʔimma* has not been entirely dropped, yet this may occur later. A final remark here is that the significance of the syntactic position in licensing grammaticalization has been reported in several previous studies (Le-ning and Chauncey 1993; Degand and Fagard, 2011; Jarrah and Alghazo, 2019; Jaradat 2021, among others).

## 4 Grammaticalisation of the vocative *ja:*

Generally speaking, languages do not only express the vocative meaning morphologically (i.e., many natural languages have morphological vocative markers, including bound morphemes and particles), but also deliver the vocative meaning via specific tunes (variations of pitch) that are carried and conveyed by vowels, as they have rich harmonic structure and high periodic energy (Sóskuthy and Roettger 2020). With respect to the emergence of morphological vocative markers (i.e., bound morphemes), Sóskuthy and Roettger (2020) have conducted a recent cross-linguistic study based on a corpus from 101 languages as an attempt to suggest a potential grammaticalization path to the evolution of morphological vocative markers. They argue that one of the possible pathways of vocative morphemes in many languages is ‘the morphological re-analysis of tune-driven phonetic variation that helps to carry pitch patterns’ (Sóskuthy and Roettger 2020:140). Their proposal is based on the main characteristics of vocative morphemes that are shared by many languages; that is, they have additional prosodic modulation, including vowel lengthening, stress shift and tone change, and few consonants. These properties are taken as evidence supporting their initial hypothesis that ‘the acoustic properties of tunes interact with segmental features and can shape the emergence of morphological markers.’ (Sóskuthy and Roettger 2020:140). On this basis, one could say that prosodic manipulations are a potential source of vocative bound morphemes in natural languages. These morphemes are possibly a result of the interactions between prosody (tune) and text (the vowels and consonants of speech). More precisely, a mid or low vowel that is inserted to carry the vocative chant (tune), can be grammaticalized to a vocative morpheme, such as the Hindi plural vocative marker/-e/.

With regard to (independent) vocative particles, their grammaticalization from a prosodic source is less likely to happen, as intrusive vocoids typically do not develop into a free particle (Hopper and Traugott 2003). Alternatively, Sóskuthy and Roettger (2020:151) suggest that the source of vocative particles can be conative interjections, which are basically to catch the attention and require the hearer's reaction or response, such as English *hey* and Jordanian Arabic/he:/.

With respect to the vocative *ja:*, what is its source? It is difficult to determine its source, whether it is prosodic or a conative interjection. It can be assumed that the vocative *ja:* has a prosodic source, as it has rich harmonic structure and high periodic energy, similar to what is reported in Sóskuthy and Roettger (2020). It consists of a glide (semi-vowel) and a long low vowel. This assumption dictates that this particle was a bound morpheme. Let us assume that this morpheme was the low vowel *a:*, which is cross-linguistically a very common vowel expressing vocative interpretation. Thus, it can be assumed that the glide *j* was inserted to avoid an onsetless particle in a vocative phrase-initial position. However, this assumption may not be borne out, as it is not common cross-linguistically that a free vocative marker develops from a tune-driven (morphologised) bound morpheme. Further, if this morphologisation were the case (i.e., *ja:* were the free form developed from a vocalic suffix), the vocative *ja:* could have had the freedom to occur in a post-vocative nominal position.^9^

Alternatively, can the vocative *ja:* be the output of the grammaticalization of a conative interjection? What argues against conative interjections as the source of the vocative *ja:* is that it can be followed by a conative interjection in SA, which is -*ha* in (17). If the vocative *ja:* had a conative sense, it would not have co-occurred with the conative -*ha* in the same sentence in (17), as this opposes the principle of language economy which favors dropping one of the two forms that deliver the same meaning or function. Additionally, the vocative *ja:* can host the conative glottal stop in (18a). This conative marker is added to call a distant addressee, akin to -*ha*. Nevertheless, these synchronic observations do not rule out the possibility that the vocative *ja:* had a conative sense and then lost it (i.e., it underwent full semantic bleaching to the conative sense). (CON = CONATIVE INTERJECTION).

**Figure:**
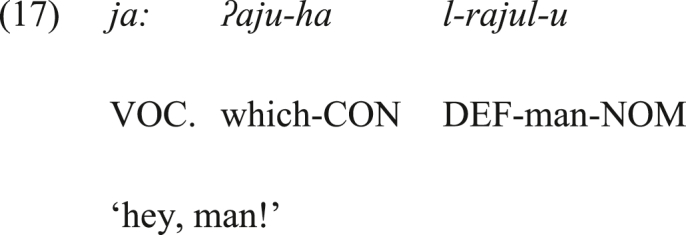

**Figure:**
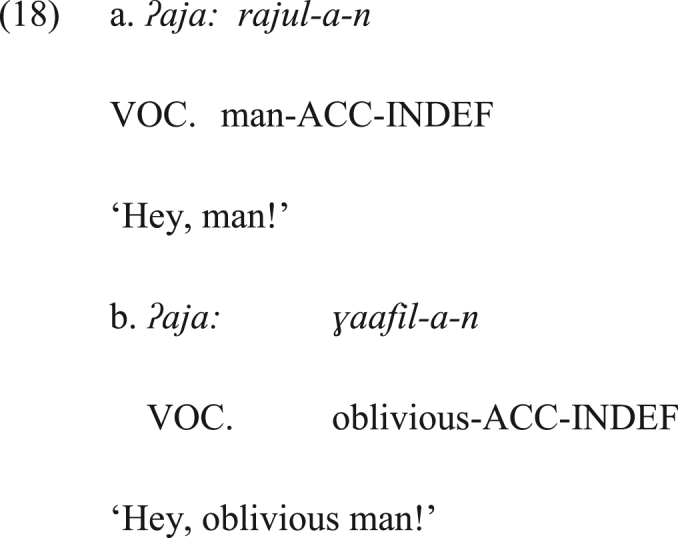

Hence, it is not plausible to propose a full grammaticalization path of the disjunctive *ja:* in the current study, as the source of the vocative *ja:* is questionable and needs more diachronic observations prior to suggesting a source the vocative *ja:*.

## 5 The disjunctive *ja:* as a case of secondary grammaticalization

Here, we argue that the development of the disjunctive coordinator *ja:* in JA is a case of secondary grammaticalization. To begin with its definition, grammaticalization is ‘a type of change whereby lexical items (such as nouns or verbs) gradually turn into grammatical items (such as auxiliaries or pronouns), after which they may continue to evolve into yet more abstract function words or even inflectional affixes. It is a reductive process, characterized by loss of semantic and phonological substance, as well as loss of syntactic freedom. Grammaticalization is therefore a ‘composite’ type of change, encompassing ‘micro-changes’ on the levels of phonology, morphology, syntax, semantics and/or discourse, either simultaneously or in succession' (Norde 2019:1).

For many researchers (Givón 1991; Norde 2019; Traugott 2002; Waltereit 2011; Smirnova 2015 among others), grammaticalization should branch into two sub-types, primary and secondary. It is commonly believed that primary grammaticalization is the shift of an item from the lexical domain (a major category) to the grammatical domain (minor category) (Hopper and Traugott 2003), such as the development the Old English lexical verb of volition *willan* ‘to want/to wish’ into the modal verb *will* that denotes futurity. On the other hand, secondary grammaticalization targets a grammatical item and makes it more grammatical, either by reducing its form or by expanding its function(s). A common example of reduction is turning the auxiliary verb *will* into the clitic *'ll*, and an example of expansion is the acquisition of the permission-denoting *may* to a new meaning, namely, possibility.

Based on the previous definition, it seems obvious that the development of the disjunctive *ja:* is not a case of primary grammaticalization, as it does not involve the shift from a major category (e.g., N, V or Adj) to a grammatical category (conjunctions/coordinators). It is rather a case of seconday grammaticalization, as the target item that underwent grammaticalization is already a grammatical item (i.e., a vocative particle). Moreover, the sub-type of secondary grammaticalization that can be observed in the case of the disjunctive *ja:* is expansion in functionality with no reduction in form (no phonological reduction) (see Detges and Waltereit 2002; Kranich 2010; Waltereit 2011; Breban 2014 for further details of secondary grammaticalization as expansion in functionality). More specifically, the grammaticalization of *ja:* results in acquiring a new function which is coordination, as they share some semantic and functional ground (the denotation of proximity and the function of warning), whilst the phonological form of *ja:* is preserved (e.g., no distressing or vowel shortening). This implies that the grammaticalization of the vocative *ja:* into a disjunctive coordinator is accompanied by semantic bleaching (or desemanticization), yet partial. The vocative meaning of *ja:* is no longer available in the disjunctive counterpart; however, the denotation of nearness or proximity and the function of warning are still there.

Additionally, the development of the vocative particle into a disjunctive coordinator is characterized by submission to more syntactic restrictions. The vocative phrase of *ja:* can appear in various places in the surface structure of a sentence, as it is not argumental (i.e., it is not an integral part of the sentence), as shown in (19).

**Figure:**
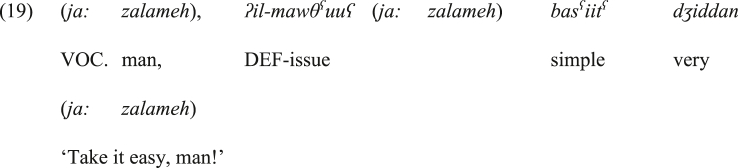

On the contrary, the disjunctive *ja:* can only appear at the initial position of each conjunct:

**Figure:**
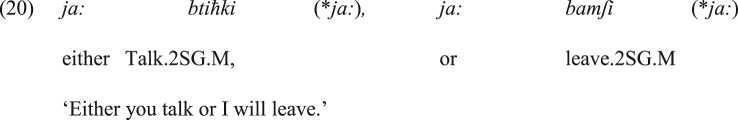

This syntactic restriction is a common secondary grammaticalization-internal process, so-called syntactic boundedness. Boundedness here is not morphosyntactic or even phonological (i.e., it does not refer to the development of bound morphemes from free morphemes). It is purely syntactic. It means that the presence of a free morpheme becomes more contingent on the surroundings/the hosted sentence. It is a result of the decategorialization of the vocative *ja:* and its re-categorialization into a disjunctive coordinator. To wrap up, the development of the disjunctive *ja:* is a case of secondary grammaticalization involving expanding its functionality and increasing its syntactic contingence on the surroundings.

## 6 Conclusion

In this research paper, it has been demonstrated that the vocative particle *ja:* has acquired a disjunctive function in a bisyndetic emphatic construction (i.e., it is a bisyndetic disjunctive coordinator) in JA. What supports the acquisition of *ja:* to the disjunctive function is that this function is conveyed by this particle in some but not all Arabic varieties, whereas the vocative function is the common cross-dialectal function of *ja:*. Further, this study suggests that the factors that licensed the development of this function (i.e., the disjunctive *ja:*) in JA are (i) the common semantic feature between them, namely proximity, (ii) the shared function of warning and (iii) the syntactic distribution of *ja:* in initial position of the two conjuncts of the bisyndetic disjunctive construction. It has also been suggested in this study that the syntactic distribution of the vocative *ja:* triggered the grammaticalization of the disjunctive *ja:*. It co-existed with *ʔimma* in conjunct-initial position. This resulted in the inheritance of the disjunctive function of *ʔimma*. This implies that the bisyndetic disjunctive construction *ja: X, ja: Y* replaced the older variant *ʔimma X, ʔu ʔimma Y*, and in between creating a stage of real superfluousness in this context, until *ja: X, ja: Y* becomes more dominant, as it currently is. Then, the superfluous structure is predicted to be dropped. It has also been demonstrated in this study that the development of the coordinating *ja:* is a case of secondary grammaticalization featured by expansion in functionality and increase in its syntactic contingence on the surroundings. From a cross-linguistic perspective, this implies that it is possible that vocatives can be a source of grammaticalized coordinators.

## Declarations

### Author contribution statement

Abdulazeez Ahmad Jaradat: Conceived and designed the experiments; Performed the experiments; Analyzed and interpreted the data; Wrote the paper.

Mohammad Anwar Al-Taher: Contributed reagents, materials, analysis tools or data.

### Funding statement

This research did not receive any specific grant from funding agencies in the public, commercial, or not-for-profit sectors.

### Data availability statement

Data included in article/supplementary material/referenced in article.

### Declaration of interests statement

The authors declare no conflict of interest.

### Additional information

No additional information is available for this paper.

## Footnotes

1 Conjuncts are coordinated phrases or sentences.

2 In the relevant literature, coordinators are also called conjunctions and coordinating particles.

3 Vocatives are typically phrases that consist of a vocative particle and a nominal (Zwicky 1974). They are basically to catch the attention (i.e., call) or to maintain the contact with the addressee (Schegloff 1968).

4 These two constructions share various properties. To exemplify some, they coordinate a wide variety of constituents, including NPs, PPs, AdjPs and full sentences. At the discourse level, it seems that they perform the same function. They emphasize that the hearer should consider each conjunct separately. However, they may exhibit some subtle differences, such as the obligatory occurrence of the conjunction *wa* to the left of the second *ʔimma*, which is ungrammatical to appear in the JA construction.

5 Arab grammarians conclude that this adverbial *ʔimma* has a number of possible interpretations, including but not limited to disjunctive interpretation (offering alternatives/options), casting doubts, giving permission (for more details of the functions and interpretations of *ʔimma* in Standard Arabic see Al-Saidawi 1999).

6 Note that Arabic has a construction of negative bisyndetic emphatic coordination, namely, *la X wala Y*, which is akin to the negative English *neither X nor Y* (Alruwaili and Sadler 2018), as shown in the following example from Turaif Arabic, which is a Saudi Arabic variety spoken in Turaif, a town in Northern Borders Province of Saudi Arabia.

7 Here, it should be noted that *ja:* at the left of the first conjunct is a scope indicator that is equivalent to English *either*, and the one at the left of the second conjunct is a disjunctive coordinator similar in meaning to *or* (Abdelhady, 2021).

8 Note that the presence of the nominative marker at the right of the nominal (the addressee) in Standard Arabic indicates that the speaker is calling (the attention) or addressing a person he can see (i.e., he is known for the speaker). On the contrary, the suffixation of the indefinite article instead means that the speaker is addressing any man (Al-Bataineh 2020).

9 The most common grammaticalization path is lexical word → free grammatical word → clitic → affix (Hopper and Traugott 2003). Note that affixes can be derivational or inflectional. As far as we can tell, the former is more likely to be the source of the latter. However, the opposite direction is possible. To exemplify, the Arabic inflectional -*at* has developed some derivational functions found in the current form of Jordanian Arabic (Jarrah et al., 2021).
